# Longitudinal assessment of HCV core antigen kinetics to monitor therapeutic response in the age of DAAs

**DOI:** 10.1371/journal.pone.0282013

**Published:** 2023-02-17

**Authors:** Suresh Ponnuvel, Arul Prakash, Runal John Steve, George Priya Doss, Ashish Goel, Uday George Zachariah, Chundamannil Eapen Eapen, Grace Rebekah, Rajesh Kannangai, Gnanadurai John Fletcher, Priya Abraham

**Affiliations:** 1 Department of Clinical Virology, Christian Medical College, Vellore, India; 2 Department of Integrative Biology, Vellore Institute of Technology, Vellore, India; 3 Department of Hepatology, Christian Medical College, Vellore, India; 4 Department of Biostatistics, Christian Medical College, Vellore, India; Centers for Disease Control and Prevention, UNITED STATES

## Abstract

**Background:**

In the economy of therapeutic monitoring, an affordable viral marker is essential in the era of direct-acting antivirals (DAAs). We elucidated the kinetics of HCVcAg to delineate its precise role in monitoring therapeutic response.

**Methods:**

In this longitudinal study, 3208 patients were tested for HCV RNA. A total of 423 patients were started on DAAs. Treatment response and kinetics of HCVcAg/RNA were assessed in treatment-naïve (n = 383) and previously treated (n = 40) patients with follow-up for 2 years.

**Results:**

After the initiation of DAAs, the rate of relapse was significantly higher in the previously treated group than naive group [12.5% (5/40) Vs 2% (7/383), p<0.0001]. The response rate at RVR was significantly higher with HCVcAg than RNA in both groups (p<0.02). The kinetics of HCVcAg and RNA were significantly different at ETR and SVR12 in the naïve (p<0.04), but similar at all therapeutic points in the previously treated group. The correlation between HCVcAg and RNA was good at baseline, ETR and SVR, except RVR in both groups (r>0.6; p<0.0001). Furthermore, HCV genotypes, treatment regimen, CTP (<7/≥7) and MELD (<15/≥15) did not influence the therapeutic response and the viral replication kinetics (p>0.05).

**Conclusions:**

It is the first longitudinal study from India shows that the response rate and kinetics of HCVcAg are comparable to HCV RNA for an extended duration, except at RVR, irrespective of the HCV genotypes, treatment regimen, and liver disease severity. Hence, HCVcAg can be considered as a pragmatic marker to monitor therapeutic response and predict relapse in the era of DAAs.

## Introduction

The hepatitis C virus (HCV) is a major public health issue, with 71 million people worldwide chronically infected. Patients with chronic HCV are more likely to develop hepatic fibrosis, cirrhosis, and hepatocellular carcinoma. HCV is an enveloped virus in the *Flaviviridae* family with a 9.6 kb ss-RNA genome. Phylogenetically, HCV is divided into 7 genotypes and 66 subtypes worldwide. Genotype 1 (44%) is the most common, followed by genotype 3 (25%) of global infections [1]. Genotype 3 (63%) predominates in India, followed by genotypes 1 (25%) and 4 (7.5%) [2].

Current treatment with highly potent direct-acting antiviral (DAA) regimens, sofosbuvir/velpatasvir (SOF/VEL), sofosbuvir/ledipasvir (SOF/VEL), sofosbuvir/daclatasvir (SOF/DCV), glecaprevir/pibrentasvir (GLE/PIB), sofosbuvir/velpatasvir/voxilaprevir (SOF/VEL/VOX) has significantly increased antiviral response rates. DAAs target various points of the HCV replication cycle, binding directly to components of the replicase complex or initiating RNA chain termination. These DAA regimens deliver a higher sustained virological response (SVR) rate of >90%, good tolerability, a higher resistance barrier, and shorter treatment duration in different genotypes [3, 4]. The pan-genotypic DAAs (SOF+VEL) could simplify care and facilitate treatment expansion worldwide. They obviate the need for HCV genotype testing prior to treatment. A small proportion of patients do not achieve SVR with DAA regimens due to the emergence of resistance associated substitutions (RAS) at baseline and the time of relapse. It can be associated with treatment failure depending upon the genotype [5, 6].

The proximate target of HCV treatment is to achieve SVR, defined by undetectable HCV RNA 12 weeks after cessation of therapy. HCV RNA quantification is a gold standard to confirm HCV infection and to monitor viral response due to its high sensitivity and specificity. However, its complexity, cost, sophisticated infrastructure, and high turnaround time can constrain its use, especially in resource-limited settings. Hepatitis C virus core antigen (HCVcAg) is an internal capsid phosphoprotein comprising 191 amino acids and is a highly conservative, antigenic, stable marker of active HCV infection. It exists in both complete virions and RNA-free proteins. HCVcAg can identify active HCV infection and is an alternative to HCV RNA [7]. It correlates with HCV-RNA and also has good comparable analytical sensitivity (80–98%) and specificity (98%) [8–10]. In patients treated with newer DAAs, HCVcAg showed a high positive predictive value for SVR [11]. Currently, there is a paucity of HCVcAg data on long-term follow-up after the initiation of DAA therapy. The European Association for the Study of the Liver (EASL) 2016 guidelines [12] and World Health Organization (WHO) Global Hepatitis Report 2017 [13] included HCVcAg testing as an alternative to HCV RNA for confirmation of HCV diagnosis. There is little data to support the use of HCVcAg testing in a diagnostic context. A cheaper and simpler diagnostic tool and follow-up during DAA therapy are urgently needed to enhance the global efforts for HCV eradication by the year 2030 [14]. Hence, this study was undertaken to elucidate the precise monitoring role of HCVcAg and compare its kinetics with HCV RNA for an extended period of time on DAA therapy in HCV infected patients.

## Methods

### Study design and patients

10 ml of EDTA blood samples were collected from the patients (n = 3208) after their written informed consent from April 2016 to March 2020 for HCV RNA quantification. The samples were centrifuged at 2500 rpm for 10 minutes for plasma separation and stored at -80°C. In this longitudinal study (n = 423), HCV RNA and HCVcAg were measured at baseline, 1m, 3m, 6m, 9m, 12m and >1 to ≤ 2 years after starting DAA treatment (SOF+DCV, SOF+LDV, SOF+VEL, SOF+RBV) for 12 or 24 weeks. Rapid virological response (RVR), end-of-treatment response (ETR), and sustained virological response (SVR) were assessed using HCVcAg and HCV RNA. Previous treatment history, ALT, AST, total bilirubin, total protein, albumin, platelet count, prothrombin time, International Normalized Ratio (INR), serum creatinine, and liver disease stages such as Child-Turcotte-Pugh (CTP), model for end-stage liver disease (MELD) scores were also taken. This study was approved by the Institutional Review Board of Christian Medical College, Vellore (IRB min. no: 14426).

### HCV RNA quantification

HCV RNA was quantified from plasma samples by using automated real-time PCR (Abbott Real-Time HCV RNA assay; Abbott Molecular, USA) with a dynamic range of quantification of 12 to 10^8^ IU/mL.

### HCV genotyping

HCV RNA was extracted from plasma samples using the QIAamp Viral RNA mini kit (Qiagen, Germany) as per the manufacturer’s protocol. The NS5B region was sequenced using hemi-nested PCR. The Titanium^®^ One-Step RT-PCR kit was used for the first round of PCR, and Supertherm Taq DNA polymerase (Medox^®^) was used for the second round. The amplified 392 bp product from the second-round PCR was sequenced after a clean-up step to remove primer dimers and excess dNTPs. DNA sequencing was performed through the ABI genetic analyzer 3500 and the results were analysed by BioEdit sequencing alignment software and submitted to the HCV BLAST sequence database to obtain the genotype results. The primer sequences are shown in S1 Table [15].

### HCVcAg quantification

HCVcAg (ARCHITECT HCV Ag, Abbott, Wiesbaden, Germany) quantification was performed in the stored plasma samples. To avoid any potential debris blocking the Architect sample aspiration needle, plasma samples were centrifuged at 7000 rpm for 7 minutes and transferred (200 μL) into 2 mL sample cups. The HCVcAg assay is a two-step chemiluminescent microparticle immunoassay (CMIA) that quantifies HCVcAg using microparticles coated with monoclonal anti-HCV. The first step of pretreatment lyses the viral particles and extracts the HCVcAg. In the second step, any HCV core antigen present in the pretreated sample binds to the anti-HCV coated microparticles conjugated with acridinium-labeled anti-HCV conjugate. The resulting chemiluminescent reaction is measured as relative light units (RLUs). The concentration of HCVcAg in each specimen was determined using an Architect HCV Ag calibration curve generated during the assay calibration. The linearity of the HCVcAg assay is 3 to 20000 fmol/L. The assay cut-off threshold for a positive result was ≥3.0 fmol/L, whereas values of 3.0 to 10 fmol/L and >10 fmol/L were reported as weak positive and positive, respectively. The weak positive samples (3.0 to 10 fmol/L) were repeated in duplicates as per the recommendation of the manufacturer.

### Conversion factor and assessment of efficacy

In this study, HCVcAg concentration in fmol/L was converted into IU/mL using a conversion factor of 1 fmol/L = 500 IU/mL (2.7 log/IU/mL) to visualize the difference between HCV RNA and HCVcAg as used in previous studies [16]. The therapeutic efficacy in the treatment of HCV infection is assessed by the following; rapid virological response (RVR)-undetectable HCV RNA or non-reactive HCVcAg at week 4 after initiation of treatment, end of treatment response (ETR)-undetectable HCV RNA or non-reactive HCVcAg at the time of completion of treatment, sustained virological response 12–74 (SVR12-74)-undetectable HCV RNA or non-reactive HCVcAg after respective 12–74 weeks of completion of treatment [17].

### Statistical analysis

All statistical analysis was performed using log 10 transformations of HCV RNA (IU/mL) and HCVcAg (IU/mL) as inputs. Clinical data and proportions were presented as mean with a 95% confidence interval and compared using the student’s independent samples t-test. A correlation coefficient and linear regression analysis were performed to assess the association between HCVcAg and HCV RNA levels. MedCalc^®^ v20.112 was used for all statistical analysis, and a p-value of <0.05 (two-tailed) was considered statistically significant.

## Results

### Patient demographics

**Fig 1:**
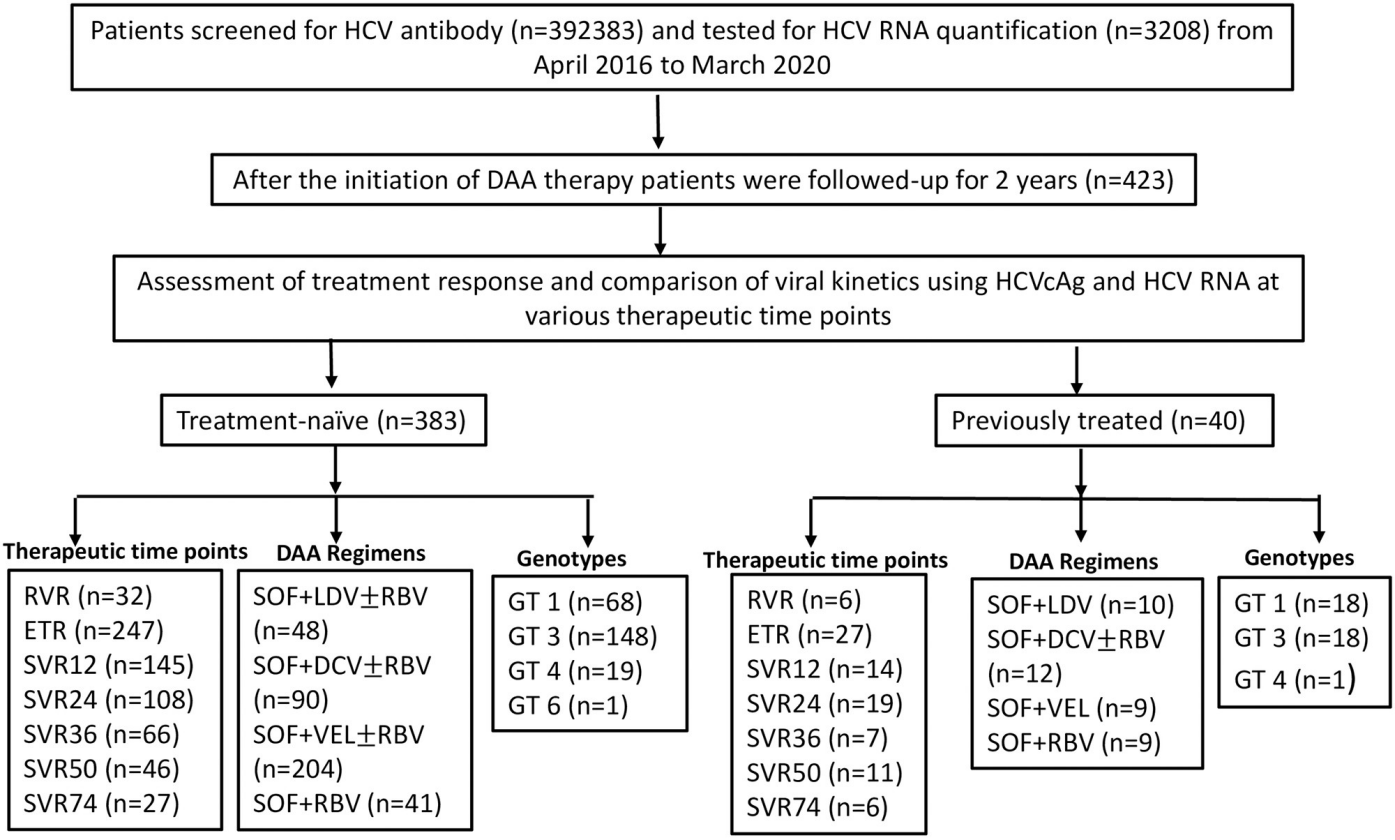
Flow chart of the study. Abbreviations: HCV, hepatitis C virus; RNA, ribonucleic acid; n, number; HCVcAg, hepatitis C virus core antigen; DAA, direct acting antiviral; RVR, rapid virological response; ETR, end of treatment response; SVR, sustained virological response; SOF, sofosbuvir; LDV, ledipasvir; DCV, daclatasvir; VEL, velpatasvir; RBV, ribavirin; GT, genotype.

The patient type was categorized into treatment-naive and previously treated patients. The baseline demographic, clinical and virological characteristics of all 423 enrolled patients are shown in Table 1, and the flow-chart of the study is shown in Fig 1.

**Table 1:** Baseline demographic, clinical and virological characteristics of 423 study patients.

Parameter(s)	Treatment-naïve (n = 383)		Previously treated (n = 40)	
	n (%)	Mean concentration (95% CI)	n (%)	Mean concentration (95% CI)
Age	383 (100)	47 (45–48)	40 (100)	45 (41–49)
Male	223 (58)	-	28 (70)	-
Female	161 (42)	-	12 (30)	-
Portal hypertension	44 (11)	-	7 (18)	-
Ascites	42 (11)	-	3 (8)	-
Hepatomegaly	39 (10)	-	4 (10)	-
Hepatic encephalopathy	3 (1)	-	0 (0)	-
HCV/HBV co-infected	19 (5)	-	1 (3)	-
HCV/HIV co-infected	6 (2)	-	1 (3)	-
Alcohol consumers	12 (3)	-	0 (0)	-
SOF+LDV±RBV	48 (13)	-	10 (25)	-
SOF+DCV±RBV	90 (23)	-	12 (30)	-
SOF+VEL±RBV	204 (53)	-	9 (22.5)	-
SOF+RBV	41 (11)	-	9 (22.5)	-
Treatment duration (12 weeks)	249 (65)	-	13 (33)	-
Treatment duration (24 weeks)	134 (35)	-	27 (68)	-
Genotype 1	68 (18)	-	18 (45)	-
Genotype 3	148 (39)	-	18 (45)	-
Genotype 4	19 (5)	-	1 (3%)	-
Genotype 6	1 (0)	-	0 (0)	-
CTP A (5–6)	221 (58)	5.19 (5.14–5.24)	23 (58)	5.08 (4.96–5.21)
CTP B (7–9)	60 (58)	7.36 (7.2–7.52)	6 (15)	7 (7–7)
CTP C (10–15)	4 (1)	10.5 (9.58–11.41)	1 (3)	11
CTP B & C (7–15)	64 (17)	7.56 (7.31–7.8)	7 (18)	7.57 (6.17–8.96
MELD <15	255 (67)	8.42 (8.14–8.71)	25 (63)	8.52 (7.66–9.37)
MELD ≥15	39 (10)	18.94 (18–19.85)	3 (8)	21.33 (2.03–40.62)
Baseline HCV RNA (log IU/mL)	383 (100)	5.52 (5.43–5.61)	40 (100)	5.62 (5.3–5.94)
Baseline HCVcAg (log IU/mL)	383 (100)	5.21 (5.12–5.31)	40 (100)	5.12 (4.76–5.48)
Total bilirubin (mg/dL)	372 (97)	1 (0.92–1.1)	36 (90)	1.04 (0.75–1.33)
Total protein (g/dL)	370 (97)	7.66 (7.21–8.12)	36 (90)	7.27 (7.02–7.52)
Albumin (g/dL)	370 (97)	3.97 3.89–4.04)	36 (90)	3.89 (3.68–4.11)
ALT (U/L)	372 (97)	52 (62–75)	32 (80)	62 (49–75)
AST (U/L)	361 (94)	69 (63–75)	30 (75)	58 (45–71)
Serum Creatinine (mg/dL)	373 (97)	1.46 (1.21–1.71)	39 (98)	1.13 (0.58–1.67)
Platelet count (Cells/μL)	360 (94)	179838 (168164–191513)	37 (93)	157081 (129361–184800)
Prothrombin time (Seconds)	301 (79)	12.73 (12.33–13.13)	30 (75)	12.69 (11.35–14)
INR	301 (79)	1.09 (1.06–1.11)	30 (75)	1.11 (1.03–1.2)

Abbreviations: n, number; CI, confidence interval; HCV, hepatitis C virus; RNA, ribonucleic acid; HCVcAg, hepatitis C virus core antigen; HBV, hepatitis B virus; HIV, human immunodeficiency virus; SOF, sofosbuvir; LDV, ledipasvir; DCV, daclatasvir; VEL, velpatasvir; RBV, ribavirin; CTP, child-turcotte-pugh; MELD, model for end stage liver disease; ALT, alanine transaminase; AST, aspartate transaminase; INR, international normalized ratio.

### Overall treatment response rate and kinetics in treatment-naïve patients

**Fig 2:**
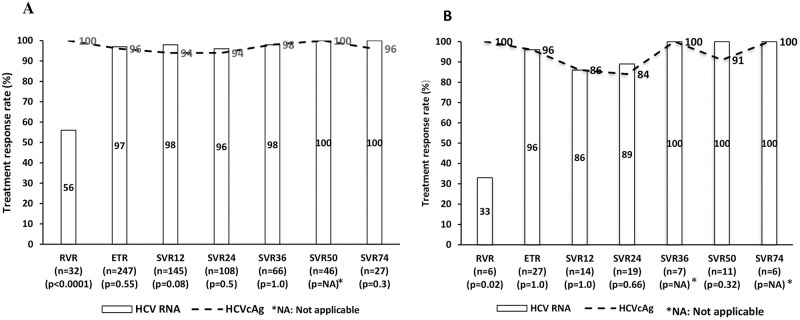
Treatment response rate assessed by HCV RNA & HCVcAg at various time points. **(A)Treatment-naïve (n = 383).** Mean HCVcAg levels (log IU/mL) in HCV RNA negative individuals at ETR (n = 6), SVR12 (n = 5), SVR24 (n = 2) & SVR74 (n = 1) are 3.8, 3.7, 3.5 & 3.39 respectively. **(B) Previously treated (n = 40).** HCVcAg levels (log IU/mL) in HCV RNA negative individuals at SVR24 (n = 1) & SVR50 (n = 1) are 4.1 & 3.46 respectively. Abbreviations: HCV, hepatitis C virus; HCVcAg, hepatitis C virus core antigen; RVR, rapid virological response; ETR, end of treatment response; SVR, sustained virological response.

**Fig 3:**
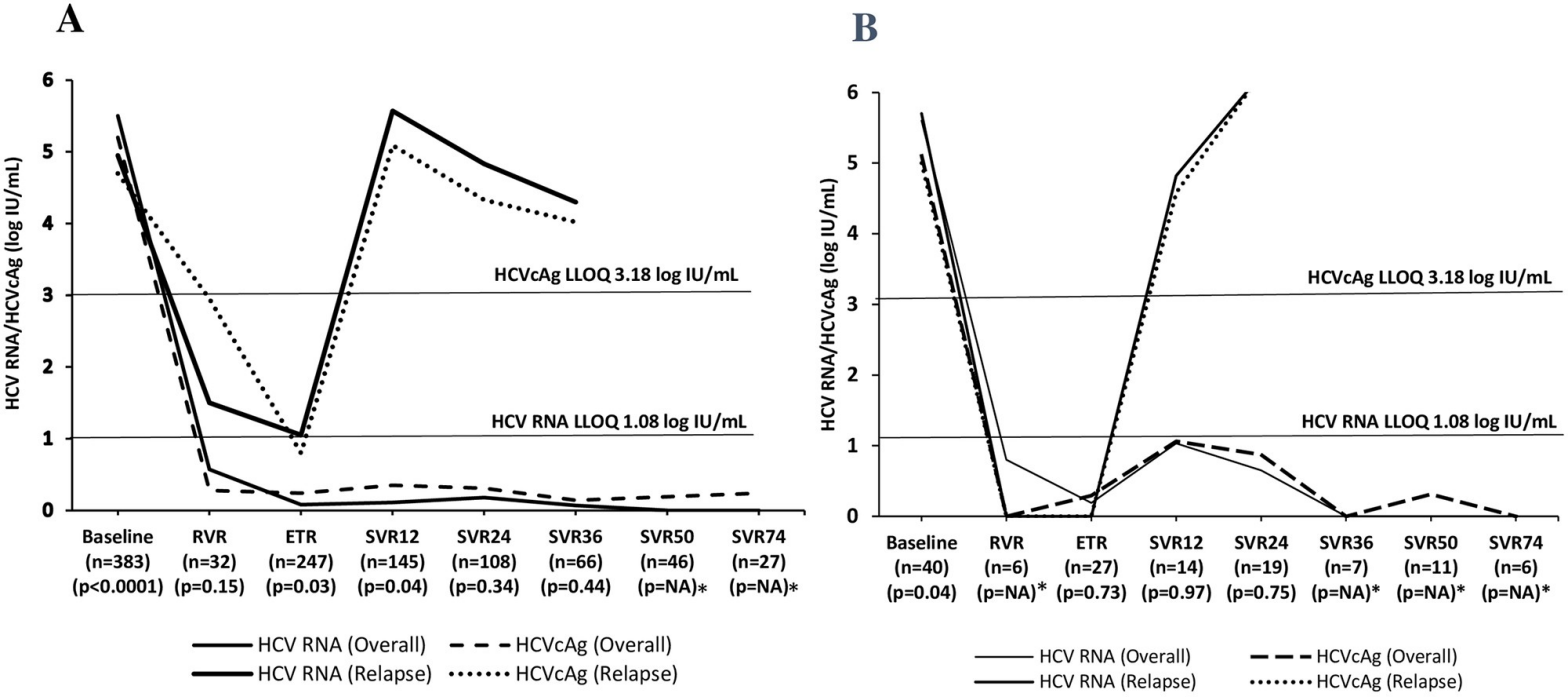
Kinetics of HCV RNA & HCVcAg at various time points. **(A) Treatment-naïve (n = 383). (B) Previously treated (n = 40).** Abbreviations: HCV, hepatitis C virus; HCVcAg, hepatitis C virus core antigen; LLOQ, lower limit of quantification RVR, rapid virological response; ETR, end of treatment response; SVR, sustained virological response; *NA: not applicable.

**Fig 4:**
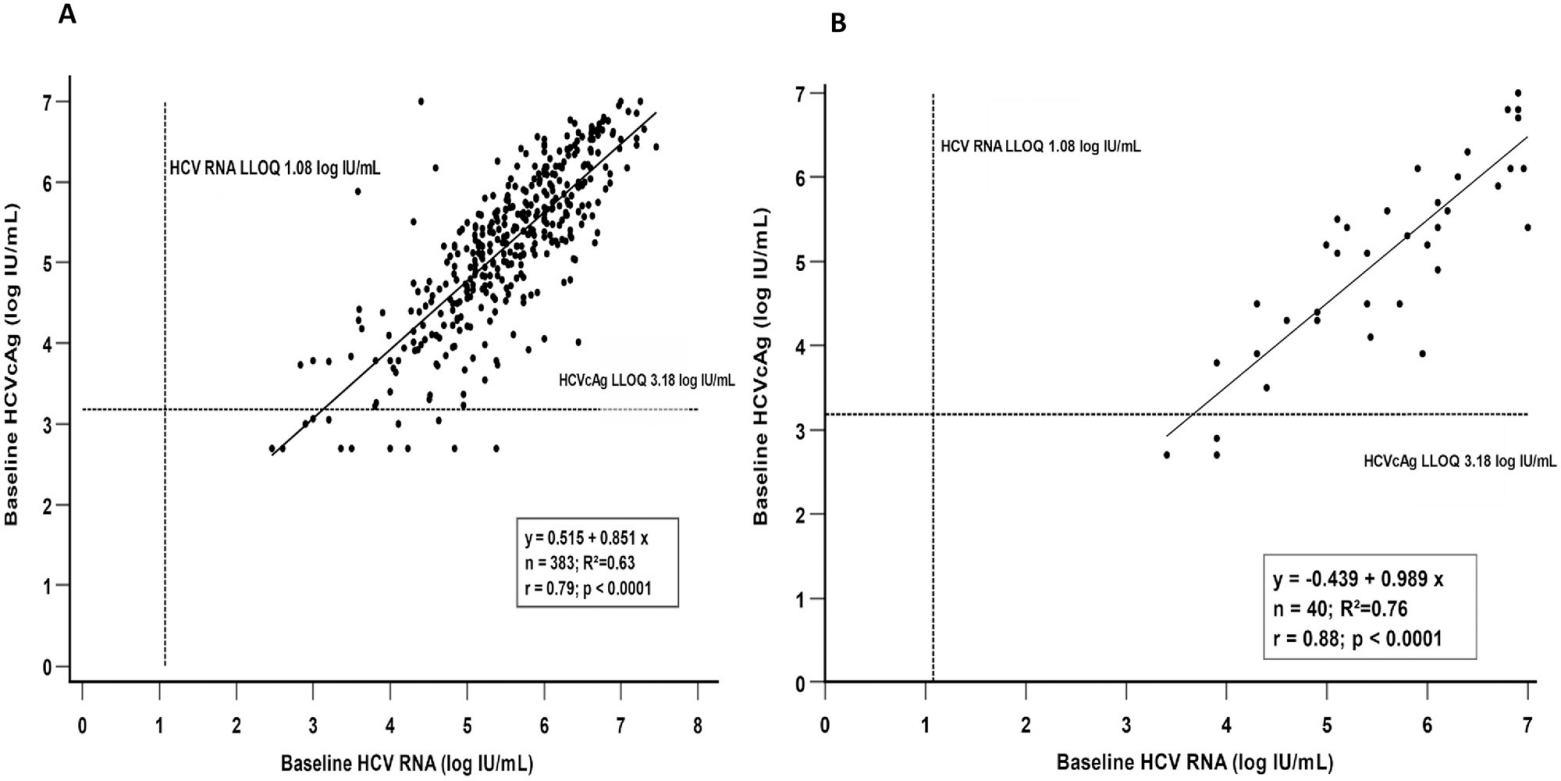
Baseline correlation between HCV RNA and HCVcAg. **(A) Treatment-naïve. (B) Previously treated.** Abbreviations: HCV, hepatitis C virus; RNA, ribonucleic acid; HCVcAg, hepatitis C virus core antigen; LLOQ, lower limit of quantification.

Among the treatment-naive patients (n = 383), the response rates between HCV RNA and HCVcAg at RVR, ETR, and SVR12-74 were 56%, 97%, >98%, and 100%, 96%, >94%, respectively (Fig 2A). In addition, among various therapeutic time points, only the RVR rate was significantly higher with HCVcAg than with HCV RNA (100% Vs 56%; p<0.0001). Similarly, the mean HCV RNA/HCVcAg concentrations at baseline, RVR, ETR, and SVR12-74 were 5.5/5.2, 0.57/0.28, 0.08/0.24, and >0.07/0.14 log IU/mL. The kinetics of HCVcAg and HCV RNA were significantly different at baseline, ETR, and SVR12 except at RVR (p<0.04) (Fig 3A). Among the 383, 7 (2%) patients who had relapse, the mean HCV RNA/HCVcAg concentrations at baseline, RVR, ETR, and SVR12-36 were 5.2/4.88, 1.5/2.95, 2.44/2.37, and >4.3/4.02 (Fig 3A). Viral replication kinetics inferred by HCV RNA and HCVcAg concentrations showed no significant difference at therapeutic time points. The correlation between HCVcAg and RNA was good at baseline, ETR, and SVR12-36, except at RVR (r>0.6; p<0.0001) The baseline correlation between HCV RNA and HCVcAg is shown in Fig 4A.

### Treatment response and kinetics based on various DAA regimens in treatment-naive patients

**Fig 5:**
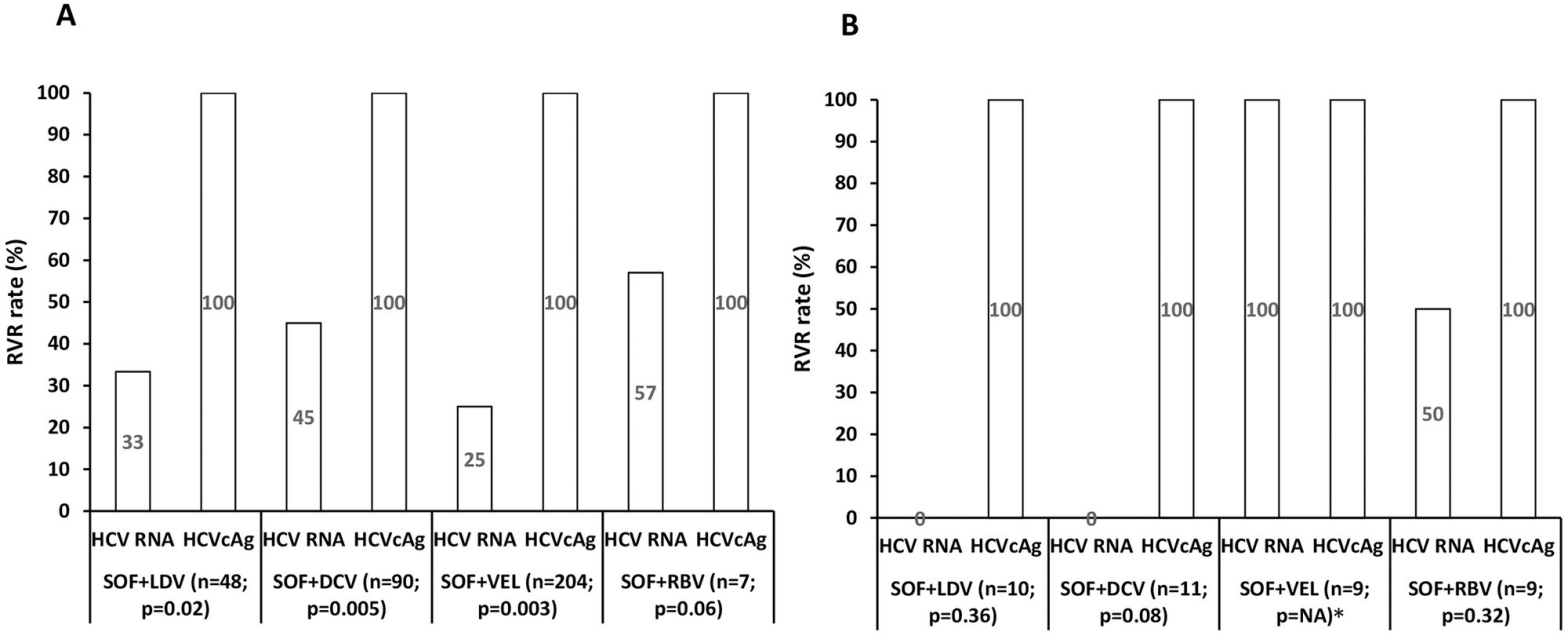
RVR rate in various DAA regimens (A) Treatment-naïve. (B) Previously treated. Abbreviations: HCV, hepatitis C virus; RNA, ribonucleic acid; HCVcAg, hepatitis C virus core antigen; RVR, rapid virological response; SOF, sofosbuvir; LDV, ledipasvir; DCV, daclatasvir; VEL, velpatasvir; RBV, ribavirin; *NA: not applicable.

Among the patients who were started on various regimen the response rate assessed by HCV RNA/HCVcAg were significantly different only at RVR for SOF+LDV (33/100%; p = 0.02), SOF+DAC (45/100%; p = 0.005), SOF+VEL (25/100%; p = 0003), SOF+RBV (57/100% p = 0.06). There was no significant difference in concentrations between these replicative markers during follow-up (Fig 5A).

### Treatment response rate and kinetics in treatment-naive patients of various genotypes

**Fig 6:**
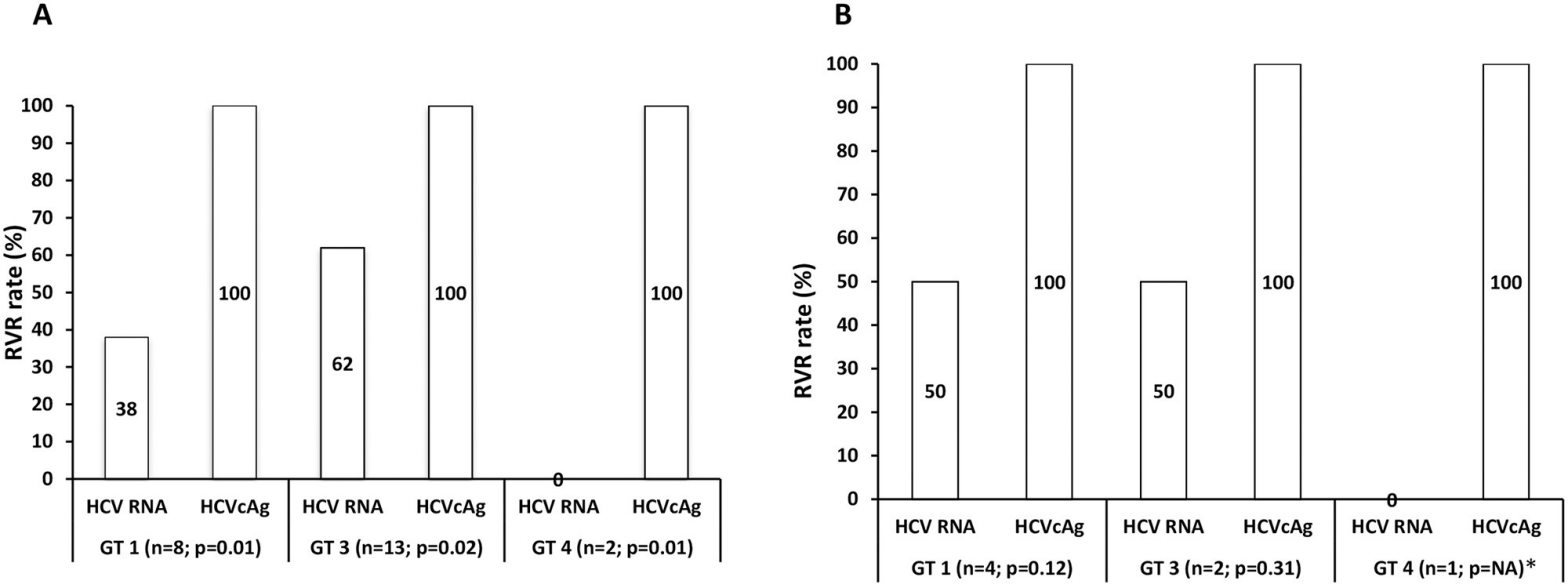
RVR rate in various genotypes (A) Treatment-naïve. (B) Previously treated. Abbreviations: HCV, hepatitis C virus; RNA, ribonucleic acid; HCVcAg, RVR, ribonucleic acid; hepatitis C virus core antigen; RVR, rapid virological response; GT, genotype; *NA: not applicable.

**Fig 7:**
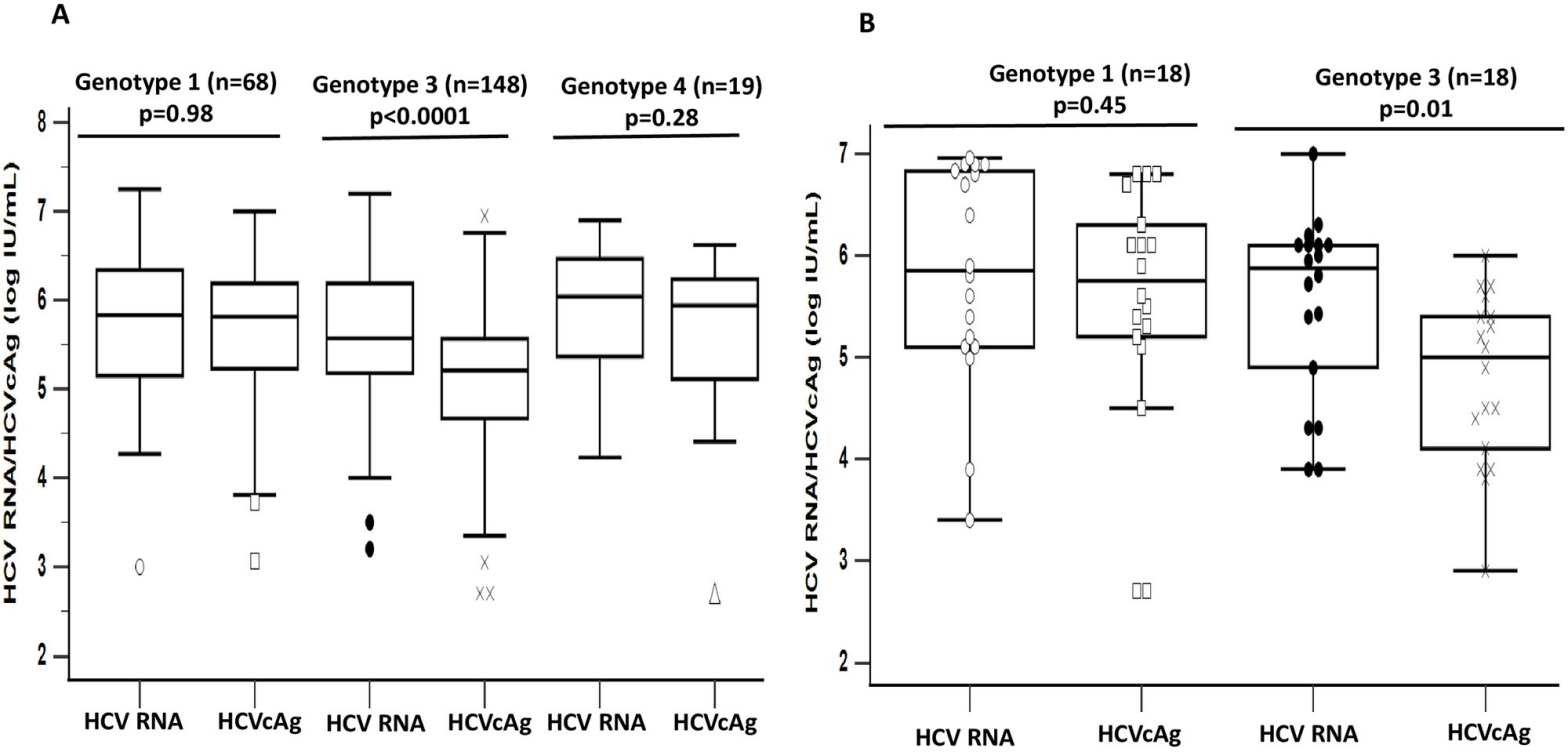
Baseline HCV RNA and HCVcAg levels in various genotypes (A) Treatment-naïve (B) Previously treated. Abbreviations: HCV, hepatitis C virus; RNA, ribonucleic acid; HCVcAg, hepatitis C virus core antigen.

The genotype frequencies in these patients were genotype 3 (39%), genotype 1 (18%), and genotype 4 (5%). Among the patients with genotype 1, 3, and 4, the response rate assessed by HCV RNA/HCVcAg was significantly different only at RVR for GT1 (38/100%; p = 0.01), GT3 (62/100%; p = 0.02), and GT4 (0/100%; p = 0.01) (Fig 6A). In addition, the baseline concentration between HCV RNA and HCVcAg was significantly different only for genotype 3 (p<0.0001) (Fig 7A). However, there was no significant difference in concentrations between these replicative markers during follow-up.

### The influence of liver disease severity and stages on therapeutic efficacy in treatment-naive patients

**Fig 8:**
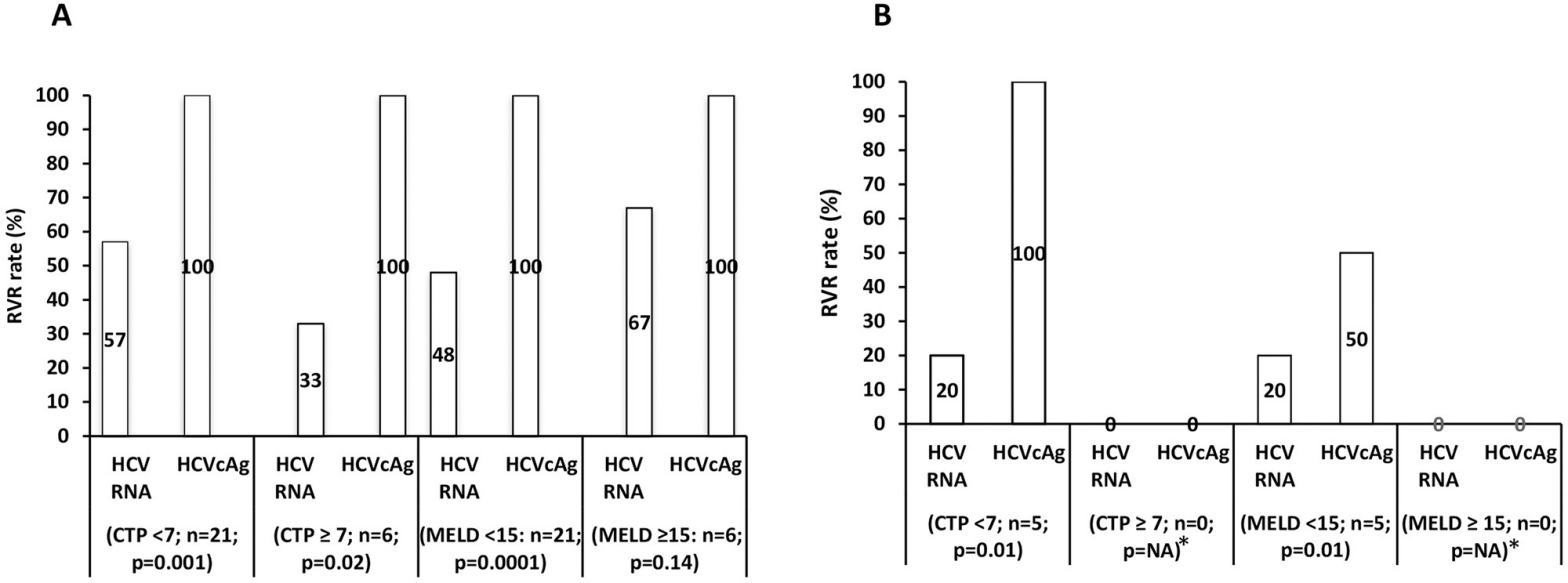
RVR rate based on CTP & MELD scores. **(A) Treatment-naïve. (B) Previously treated.** Abbreviations: HCV, hepatitis C virus; RNA, ribonucleic acid; HCVcAg, RVR, rapid virological response; hepatitis C virus core antigen; CTP, child-turcotte-pugh; MELD, model for end stage liver disease; *NA: not applicable.

The proportion of patients who had achieved RVR as assessed by HCVcAg was significantly higher than RNA irrespective of CTP score (<7, 57/100%, p = 0.001; ≥7, 33/100%, p = 0.02). However, the disease severity did not significantly alter the concentrations of viral replicative markers at all therapeutic time points. The proportion of patients who had achieved RVR as assessed by HCVcAg was significantly higher than RNA irrespective of MELD score (<15, 48/100%, p = 0.0001; ≥15, 67/100%, p = 0.14). However, the disease severity did not significantly alter the concentrations of viral replicative markers at all therapeutic time points (Fig 8A).

### Overall treatment response rate and kinetics in previously treated patients

Among the previously treated patients (n = 40), the response rates between HCV RNA and HCVcAg at RVR, ETR, and SVR12-74 were 33%, 96%, >86%, and 100%, 96%, >84%, respectively (Fig 2B). In addition, among various therapeutic time points, only the RVR rate was significantly higher with HCVcAg than with HCV RNA (100% Vs 33%; p<0.0001). Similarly, the mean HCV RNA/HCVcAg concentrations at baseline, RVR, ETR, and SVR12-74 were 5.6/5.1, 0.8/0, 0.19/0.29, and <1.03/1.06 log IU/mL, respectively (Fig 3B). The kinetics of HCVcAg and HCV RNA were significantly different only at baseline (p<0.04). Among the 40, 5(12.5%) had relapsed. The mean HCV RNA/HCVcAg concentrations were 5.7/5, 0/0, 0/0, >4.82/4.58 log IU/mL at baseline, RVR, ETR, and SVR12-36, respectively (Fig 3B). Viral replication kinetics inferred by HCV RNA and HCVcAg concentrations showed no significant difference at therapeutic time points. The correlation between HCVcAg and RNA was good at baseline, ETR, and SVR12-24 (r>0.6; P<0.0001). The baseline correlation between HCV RNA and HCVcAg is shown in Fig 4B.

### Treatment response and kinetics based on various DAA regimens in previously treated patients

Among the patients who were started on various regimens, the response rate and concentrations assessed by HCV RNA/HCVcAg were not significantly different at baseline and therapeutic follow-up (Fig 5B).

### Treatment response and kinetics in previously treated patients of various genotypes

The genotype frequencies in these patients were genotype 3 (45%), 1 (45%) and 4 (2.5%). The response rates assessed by HCV RNA and HCVcAg were not significantly different for GT1, GT3, and GT4 at all therapeutic points (Fig 6B). In addition, the baseline concentration between HCV RNA and HCVcAg was significantly different only for genotype 3 (p<0.01) (Fig 7B). However, there was no significant difference in concentrations between these replicative markers during follow-up for all genotypes.

### The influence of liver disease severity and stages on therapeutic efficacy in previously treated patients

In patients with low CTP (<7) and MELD score (<15), the proportion who have achieved RVR as assessed by HCVcAg was significantly different than RNA (20/100%, p = 0.01). However, the disease severity did not significantly alter the concentrations of these viral replicative markers at all therapeutic time points (Fig 8B).

## Discussions

Elimination of HCV by 2030 as a public health threat needs reliable and affordable direct viral markers to assess the therapeutic response in the era of DAAs [14]. Currently, HCV RNA is the gold standard in treatment monitoring. However, affordability remains an inherent challenge. HCVcAg is a direct marker of active replication. It is highly stable, appears at about the same time as HCV RNA and is emerging as a reliable and affordable alternative [18]. Nevertheless, there is a paucity of data on the long-term assessment of HCVcAg kinetics in DAAs. Currently, there are no guidelines available to assess the response using HCVcAg.

Among the treatment-naïve patients who were positive for HCV RNA, 96.6% were positive and 4.4% were negative for HCVcAg. This indicates that the HCV RNA is more sensitive as direct viral marker to initiate treatment. After the initiation of DAAs in the treatment naive and previously treated groups, the RVR rate was significantly higher when assessed by HCVcAg than by HCV RNA. This difference is due to HCV RNA’s inherent superior sensitivity (12 IU/mL = 1.08 log IU/mL) over HCVcAg (3 fmol/L = 1500 IU/mL = 3.18 log/IU/mL), with viral loads of <0.8 log IU/mL as expected [8, 19].

However, on further follow-up, the rate of response and the kinetics of HCVcAg/HCV RNA were similar at ETR and SVR12-74 even in patients with relapse. Though there were changes in HCVcAg and HCV RNA concentration, none of the therapeutic time points had >0.5 log IU/mL difference. Considering the biphasic model of viral kinetics, the baseline difference in the concentrations between HCVcAg and RNA could have resulted in transient discordance between these markers exclusively at RVR. Similar to HCV RNA at RVR, mathematical modelling on early HCVcAg kinetics can be considered to delineate its precise role in early therapeutic monitoring. Hence the management of these patients who had discrepant HCV RNA and HCVcAg levels exclusively in RVR does not require special therapeutic consideration as the final treatment outcomes were similar.

The rate of relapse as evaluated by HCVcAg/HCV RNA was similar and significantly higher in the previously treated group than in the treatment-naive group (p<0.006). It is well known that previously treated patients are known to harbour resistance associated substitutions (RAS) who are more likely to relapse on retreatment than treatment-naive [20]. However, the kinetics of HCVcAg was similar to RNA in relapsed patients in both groups. Furthermore, the presence of residual HCV RNA is not uncommon and it may not predict relapse. However, one of the limitations was loss to follow-up at various therapeutic time-points due to financial constraint and long-travel required to seek help at the level of tertiary care center.

HCV genotypes exhibit geographical restrictions and are known to differentially influence disease progression and outcome [21]. The study cohort had 3 genotypes (1, 3, and 4) with the predominance of genotype 3. However, genotypes/subtypes, liver disease severity (CTP/MELD) did not influence the rate of response and kinetics of HCVcAg/RNA at all therapeutic time points. This is in contrast to other studies which indicated the influence of CTP/MELD on the therapeutic outcome [22].

In conclusion, longitudinal data suggest that, regardless of HCV genotype, treatment regimen, or severity of liver disease, the response rate and kinetics of HCVcAg are comparable to HCV RNA for an extended period of time. In the economy of therapeutic monitoring, HCV RNA has diminishing returns because of its high cost and high response/cure rate associated with DAAs. Hence, HCVcAg can be considered as a pragmatic marker to monitor therapeutic response and predict relapse.

## Supporting information

S1 Table Primer sequences. (DOCX)

S1 File Overall data file. (XLSX)
