# Supplementary material for: Longitudinal assessment of HCV core antigen kinetics to monitor therapeutic response in the age of DAAs
Source: PLoS One. 2023 Feb 17;18(2):e0282013. doi: 10.1371/journal.pone.0282013 (PMC9937470; doi:10.1371/journal.pone.0282013)
Supplement: S1 Table — (DOCX) [file pone.0282013.s001.docx]

**Supplementary Table 1. Primer sequences.**

| **PCR** | **Primer name** | **Primer sequence (5’→3’)** | **Target region** |
| --- | --- | --- | --- |
| *First round PCR | Forward P1204 | GGAGGGGCGGAATACCTGGTCATAGCCTCCGTGAA | NS5B |
|  | Reverse P1203 | GGGTTCTCGTATGATACCCGCTGCTTTGACTC |  |
| *Second round & sequencing PCR | Forward P1204 | GGAGGGGCGGAATACCTGGTCATAGCCTCCGTGAA |  |
|  | Reverse NS5B IP | TGATACCCGCTGCTTTGACTCNACNGTCAC |  |
